# Identifying intimate partner violence: comparing the Chinese abuse assessment screen with the Chinese revised conflict tactics scales

**DOI:** 10.1111/j.1471-0528.2007.01441.x

**Published:** 2007-07-06

**Authors:** A Tiwari, DYT Fong, KL Chan, WC Leung, B Parker, PC Ho

**Affiliations:** a Department of Nursing Studies, Li Ka Shing Faculty of Medicine Hong Kong; b Department of Social Work and Social Administration, The University of Hong Kong Hong Kong; c Department of Obstetrics and Gynaecology, Kwong Wah Hospital Hong Kong; d School of Nursing, University of Virginia, Chavlottesville USA; e Department of Obstetrics and Gynaecology, Li Ka Shing Faculty of Medicine, The University of Hong Kong Hong Kong

**Keywords:** Abuse assessment screen, Chinese, intimate partner violence, revised conflict tactics scale

## Abstract

**Objective:**

To assess the measurement accuracy and the utility of the Chinese Abuse Assessment Screen (AAS).

**Design:**

A cross-sectional study.

**Setting:**

An antenatal clinic of a public hospital and a community centre in Hong Kong.

**Sample:**

A total of 257 Chinese women consisting of 100 pregnant women and 157 nonpregnant women.

**Method:**

The Chinese AAS was administered first, followed by the Chinese Revised Conflict Tactics Scales (CTS2). This was performed in the same sitting, and each participant was interviewed once either at an antenatal clinic (for the pregnant women sample) or at a community centre (for the nonpregnant women sample).

**Main outcome measures:**

Estimates of the sensitivity, specificity, positive and negative predictive values and positive and negative likelihood ratios.

**Results:**

Using the Chinese CTS2 as the standard, the specificity estimates of the Chinese AAS for emotional, physical and sexual abuse were ≥89%, while the sensitivity estimates varied from 36.3 to 65.8%. The sensitivity improved in the screening for more severe cases (66.7%). The positive predictive values were ≥80%, and the negative predictive values varied from 66 to 93%. Factors such as the age difference between the couple and the woman’s need for financial assistance were found to be associated with intimate partner violence (IPV).

**Conclusion:**

The Chinese AAS has demonstrated satisfactory measurement accuracy and utility for identifying IPV when the Chinese CTS2 was used as the standard.

## Introduction

Intimate partner violence (IPV) is a major public health problem with profound health consequences.^1^ Organisations of health professionals have recommended screening women for domestic violence in healthcare settings (e.g. the American College of Obstetricians and Gynecologists^2^ and the British Medical Association^3^). However, concern has been expressed about the lack of evidence for the effectiveness of the available screening programmes.^4^,^5^ Moreover, the accuracy of the screening tools used to identify IPV has also been questioned.^6^

Of the tools available for screening for IPV, the Abuse Assessment Screen (AAS)^7^ has been used extensively in many healthcare settings throughout the USA and internationally.^8^ Psychometric evaluations of the AAS have shown that women identified by the AAS as being abused are also found to be abused when other IPV screening tools are used, such as the Index Spouse Abuse, the Danger Assessment Screen and the Conflict Tactics Scales.^7^,^9^,^10^ However, in a recent study, which compared the results obtained using the AAS with those obtained using the Revised Conflict Tactics Scales (CTS2) with a group of women in Rio de Janeiro, Reichenheim and Moraes^11^ found that two-thirds of minor episodes and one-third of severe episodes of physical violence were not revealed by the AAS. These findings reinforce the call for validating the AAS in languages other than English.^5^

The Chinese AAS has been used to screen Chinese women for IPV and some modifications have been made based on field experience gained while using this tool.^12^–^14^ The present study has been undertaken to assess the measurement accuracy of the Chinese AAS using the Chinese CTS2 as the standard. The CTS2 is used as ‘gold standard’, as it is the most widely adopted scale for measuring prevalence, chronicity and severity of spousal conflicts.^15^ Furthermore, the Chinese CTS2 has been validated using data from the first representative household study of spousal battering in Hong Kong with satisfactory reliability and validity.^16^ Although the 39 items CTS2 is a good and reliable tool, it takes time to complete. The AAS is much shorter than the CTS2, which is useful for clinical screening.

## Methods

The data for this study were drawn from two studies to enhance the representativeness of the sample. Both studies were conducted by the authors to explore the relationship between IPV and mental health among Chinese women in Hong Kong between April 2005 and March 2006 using a cross-sectional design. The sample for the present study consisted of 257 Chinese women. Of these, 100 were pregnant women attending an antenatal clinic in a public hospital and 157 were nonpregnant women attending activities in a community centre. We deliberately included pregnant and nonpregnant women in the sample to enhance representativeness. As certain behaviours may be acceptable in nonpregnancy but unacceptable in pregnancy, a sample made up of pregnant and nonpregnant women would better ensure validity.

Constructed by the Nursing Research Consortium on Violence and Abuse,^7^ the AAS consists of questions designed to elicit the history of violence against women within a stated period of time and for the identification of the perpetrator. When used for pregnant women, the AAS addresses violence during their lifetime, during the preceding 12 months and during pregnancy. For nonpregnant women, the pregnancy question is deleted. If respondent answers ‘yes’ to the question related to the time frame of preceding 12 months or pregnancy, she will be asked to indicate the identity of the perpetrator.

The Chinese AAS used in this study is a translation of the AAS, for which the permission of the original test constructors was obtained. The Chinese AAS addresses emotional and physical violence *separately*for all time periods covered (lifetime, the preceding 12 months and during pregnancy). This differs from the English AAS, which treats emotional and physical violence simultaneously for the lifetime period, while focusing only on physical violence for the other time periods. The decision to treat emotional and physical violence separately in the Chinese AAS is justified because earlier studies have revealed that emotional abuse predominated among Chinese abused women and that many of them did not report physical violence.^12^–^14^,^16^ As emotional abuse can be subtle and there is no widely accepted definition for it,^17^ we decided to include examples of emotionally abusive behaviour in the Chinese AAS to help the respondents to assess whether it was present in their intimate relationships. The examples were drawn from our earlier studies of IPV among Chinese women in Hong Kong.^12^–^14^,^16^ Given the infinite number of tactics that could conceivably be used to emotionally abuse an intimate partner, we organised the emotionally abusive behaviours using the dimensions proposed by Maiuro,^17^ as follows:

1. Denigrating damage to self-esteem (e.g. yelling, put-downs, shaming in front of friends and family).
2. Passive-aggressive withholding of emotional support and nurturance (e.g. punitive use of avoidance, sulking and emotional abandonment).
3. Threatening behaviour (e.g. threats of physical hurt, coercive threats to take away the children and engaging in reckless driving).
4. Restricting personal territory and freedom (e.g. isolation from friends and family, stalking and controlling the use of money).

The content validity of the Chinese AAS was confirmed by a panel of seven Chinese IPV researchers consisting of three nurses, two doctors, a clinical psychologist and a social worker.

In the present study, when comparing the Chinese AAS with the Chinese CTS2, the recall time frame was strictly limited to the preceding 12 months, as the pregnancy time frame would not apply to all the respondents. Thus, only the following three questions in the Chinese AAS were selected for analysis:

1. Within the last year, have you been emotionally hurt by someone?
2. Within the last year, have you been physically hurt by someone?
3. Within the last year, has anyone forced you to have sexual activities?

The corresponding subscales of the Chinese CTS2 on emotional aggression, physical violence and sexual coercion were used as the standard for comparison.

A number of risk factors are consistently linked to IPV, including young age, low academic achievement, low income, marital conflict and low social capital.^1^ In this study, we also explored the relationships between the risk factors and the different types of IPV as a way of assessing the utility of the Chinese AAS for identifying women abused by their intimate partners.

The five research support staffs who administered the instruments received extensive training in the screening for IPV and were closely monitored by the principal investigator. The interviewer administered the instruments to each woman in a private area and without the presence of her intimate partner. The Chinese AAS was administered first and then the Chinese CTS2 but both during the same sitting.

The study was approved by the institutional review board of the University of Hong Kong/Hospital Authority. Participation in the study was voluntary, and each respondent gave informed consent. Confidentiality was guaranteed by assigning a code number to each completed questionnaire instead of using the respondent’s name, keeping the returned questionnaires and signed consent forms in a locked cabinet accessible only to the principal investigator, identifying respondents in the database by code numbers only and ensuring that under no circumstances would information provided by the respondents be revealed to anyone outside of the research team. Respondents who were identified as having suffered partner abuse were encouraged to seek help and were provided with the necessary information for referral. Whether the respondents sought referral or not, they would be followed up by our research staff (e.g. when the pregnant respondents attended antenatal clinic appointments or when the nonpregnant respondents used the services in the community centre). In addition, they were given telephone numbers of our research staff, which they could call whenever necessary. We took every precaution to safeguard the women’s safety by ensuring that their partners were not present at the time when they were recruited to the study or completing the questionnaires. We also advised the women to discard the information for referral if they felt that their partners might find out. Furthermore, the telephone number of the research support staff provided to the woman was disguised as one of her friend’s.

We followed the scoring method described in the *Manual for the Conflict Tactics Scales*^18^ for determining the thresholds for positivity and negativity. Response categories 0 (‘This never happened’) and 7 (‘Not in the past year, but it did happen before’) were scored as ‘no’. All other responses were scored as ‘yes’.

To assess the diagnostic accuracy and utility of using the Chinese AAS for screening women for IPV, the sensitivity, specificity, positive and negative predictive values, as well as the positive and negative likelihood ratios were computed using the Chinese CTS2 as the gold standard.^19^ The interpretation of the likelihood ratios was made following the methodology suggested by Jaeschke *et al.*^20^ To further assess the accuracy of the Chinese AAS for screening women for different degrees of severity of abuse, a CTS2 severity score was calculated separately for emotional, physical and sexual abuse. Specifically, in the case of emotional abuse, which was measured by eight items in the Chinese CTS2, the severity score was calculated as the number of items with a frequency of 1 or above in the preceding 12 months. The CTS2 severity scores for physical and sexual abuse were similarly calculated. Then, for each type of abuse, the false-positive and false-negative error rates were plotted against the different cutoff values of the CTS2 severity score used for defining a case. Furthermore, logistic regression was used to examine the effects of known risk factors of IPV when the Chinese AAS was used for the identification of cases. In this study, all estimates were accompanied by an exact 95% confidence interval, where appropriate. The Stata release 9 for Windows (StataCorp, College Station, TX, USA) and the SPSS version 11.5 (SPSS Inc., Chicago, IL, USA) were used to perform the analysis.

## Results

**Figure 1:**
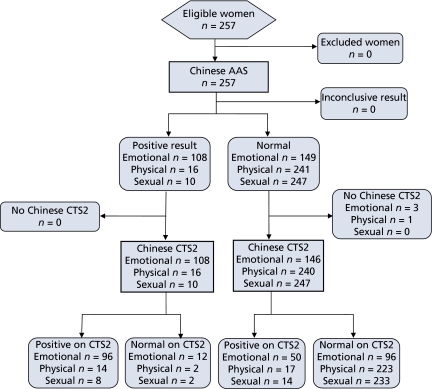
The Standards for Reporting of Diagnostic Accuracy (STARD) flowchart demonstrating the tests performed on the study population.

Figure 1 shows the tests performed on the study population. The sample of 257 Chinese women was mainly married women (91.4%, 95% CI 83.6–95.8), with a mean age of 36.2 years (SD 8.1). Compared with the general population,^21^ the individuals in the study sample are less well educated with 34.6% (95% CI 25.7–45.2) having had 9 years or less of schooling. They are also financially less well off with 44% (95% CI 34.1–54.3) having monthly family incomes lower than the official median of HK$11,000 (about US$1375).

Table 1 shows the raw agreement for the three types of abuse, reported as percentage of agreement between the Chinese AAS and the Chinese CTS2. A chance-corrected agreement, reported as kappa coefficient, is also shown. A fair agreement was found between the Chinese AAS and the Chinese CTS2.

**Table 1:** Agreement between the Chinese AAS and the Chinese CTS2

Type of abuse	*n* *	Agreement % (95% CI)	Kappa coefficient
Emotional	254	76 (66.4–83.9)	0.522
Physical	256	93 (86.1–97.1)	0.559
Sexual	257	94 (87.3–97.7)	0.472

* Number of participants who responded to the questions.

Table 2 shows the accuracy measures of the Chinese AAS concerning emotional, physical and sexual abuse in the preceding 12 months compared with the Chinese CTS2. Specificity estimates were ≥89% indicating that when the Chinese CTS2 is negative, a high percentage of the Chinese AAS were also negative. In contrast, the sensitivity estimates were much lower, especially for sexual abuse. Positive predictive values of ≥80% were encouraging, suggesting that if they were screened positive by the Chinese AAS a high percentage would also be screened positive by the Chinese CTS2. However, the negative predictive values for the different types of abuse were somewhat mixed: while those for physical and sexual abuse were ≥93%, the value for emotional abuse was only 66%. Similarly, the positive likelihood ratios were all larger than 5 indicating that the Chinese AAS is useful when it shows a positive result. However, the negative likelihood ratio for emotional abuse indicated that the Chinese AAS is only slightly useful when it shows a negative result.

**Table 2:** The accuracy of measurement when using the Chinese AAS to screen for IPV

AAS		CTS2		Se % (95% CI)	Sp % (95% CI)	PPV % (95% CI)	NPV % (95% CI)	PLR (95% CI)	NLR (95% CI)
								
		+	−						
	+	a	b						
	−	c	d						
Emotional abuse		96	12	65.8 (57.5–73.4)	88.9 (81.4–94.1)	88.9 (81.4–94.1)	65.8 (57.5–73.4)	5.92 (3.43–10.2)	0.39 (0.31–0.49)
		50	96						
Physical abuse		14	2	45.2 (27.3–64.0)	99.1 (96.8–99.9)	87.5 (61.7–98.4)	92.9 (88.9–95.8)	50.8 (12.1–213)	0.55 (0.40–0.76)
		17	223						
Sexual abuse		8	2	36.3 (17.2–59.3)	99.1 (97.0–99.9)	80.0 (44.4–97.5)	94.3 (90.7–96.9)	42.7 (9.66–189)	0.64 (0.47–0.88)
	14	233							

**Figure 2:**
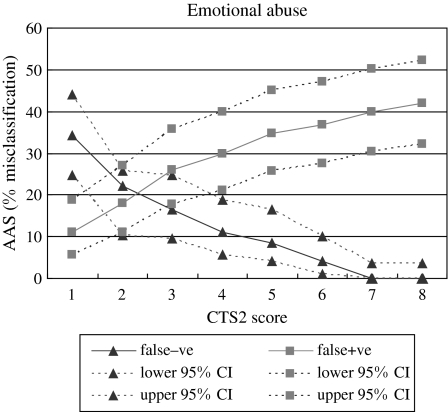
Percentages of false positives and false negatives identified by the Chinese AAS on emotional abuse.

**Figure 3:**
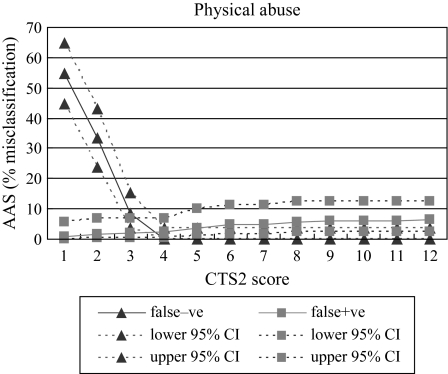
Percentages of false positives and false negatives identified by the Chinese AAS on physical abuse.

The false positives and false negatives of the Chinese AAS for screening for cases of abuse of different degrees of severity were further explored. In the case of emotional abuse, the Chinese AAS shows an upward trend of false positives but a downward trend of false negatives as the cases screened become more severe (Figure 2). For physical abuse, the Chinese AAS had a downward trend of false negatives and consistently low false positives (Figure 3). It is worth noting that despite a high false-negative level for the Chinese AAS when screening for cases with a minimum Chinese CTS2 score of 2 or below, the level improves significantly when the minimum score is 3 or above. In particular, when the minimum CTS2 score for defining a case of abuse was set at 3, the sensitivity reached 66.7% (95% CI 63.7–69.6). The corresponding specificity was 98.3% (95% CI 92.9–99.8), the positive predictive value was 75% (95% CI 65.3–83.1) and the negative predictive value was 97.5% (95% CI 96.3–98.4). The false positives and false negatives for sexual abuse are not shown due to the small number of cases (*n* = 8) of sexually abused women.

Among the risk factors for IPV, the age difference between the couple and the woman’s need for financial assistance were significantly associated with emotional and physical abuse after adjusting for the place of birth, education, number of years married and number of children. Thus, the greater the difference in age between the couple, the more likely it was that the women were emotionally (OR 1.13; 95% CI 1.03–1.23) or physically (OR 1.18; 95% CI 1.07–1.29) abused by their intimate partners. Similarly, women who were in need of financial assistance were approximately eight times more likely to be emotionally abused (OR 8.99; 95% CI 1.14–71.13) and three times more likely to be physically abused (OR 4.01; 95% CI 1.05–15.40). None of the pregnant women reported physical abuse. Compared with nonpregnant women, pregnant women were less likely to report emotional abuse (OR 0.012; 95% CI 0.003–0.053).

## Discussion

The original AAS, using a series of simple questions to identify IPV, has been used in a variety of healthcare settings.^8^ Whether the purpose is for screening in clinical settings or identifying abused subjects for purposes of research, the expectation is that the AAS should be able to detect ‘true’ events of IPV or, conversely, confidently dismiss negative cases. The same is also expected of the Chinese AAS.

In the present study, the Chinese AAS has demonstrated a satisfactory level of measurement accuracy with high specificity and positive predictive values and satisfactory to high negative predictive values. The sensitivities, however, are somewhat lower. Nevertheless, the AAS appears to be a useful screening tool as reflected by the positive likelihood ratios.^20^

An earlier study comparing the effectiveness of the AAS with the CTS2 in identifying physical violence during pregnancy has raised concern about the use of the AAS as a stand-alone screening tool for IPV. It was found that the AAS failed to identify a considerable number of IPV victims.^11^ The findings of the present study, however, do not support this concern. This may be due in part to the fact that explicit examples of emotionally abusive acts have been added to the Chinese AAS, in line with the improvements to the AAS suggested by Reichenheim and Moraes.^11^

Notwithstanding the encouraging findings, further improvement of the Chinese AAS is recommended, especially in regard to sensitivity. As shown in Table 2, the Chinese AAS misses some cases of IPV, which are detected by the Chinese CTS2. The discrepancy between the instruments may be due to the way answers are elicited from respondents. In the Chinese CTS2, respondents are asked to indicate whether certain acts have taken place (e.g. in the emotional aggression subscale: ‘when we have an argument, he had insulted or swore at me’ or in the physical violence subscale: ‘he had pushed or shoved me’). In the Chinese AAS, respondents are asked to indicate if they have been emotionally or physically hurt by someone, if so, who that person is. Examples are provided to help them understand the meaning of ‘emotional hurt’ (none provided for ‘physical hurt’). It is plausible that despite the examples provided, some respondents may not consider certain acts (probably mild ones occurring at infrequent intervals) as constituting emotional abuse and therefore give a negative response to the Chinese AAS. For physical abuse, as no examples are provided in the Chinese AAS, respondents may interpret certain acts as not constituting physical abuse (although they are listed in the physical violence subscale of the Chinese CTS2). This may explain why a respondent may give a positive answer to the Chinese CTS2 but a negative answer to the Chinese AAS in the same subscale. We suggest a modified edition of the Chinese AAS with specific acts of abuse incorporated in the wording of all the questions. In this way, interpretation on the part of the respondent would be eliminated. In fact, more acts of violence have already been included in the questions in the original AAS to enhance its performance. For example, two violent acts, ‘pushing’ and ‘shoving’, have been added to the physical abuse items.^22^

The upward trend of false positives and the downward trend of false negatives for emotional abuse as shown in Figure 2 warrant further consideration. It is worth noting that the Chinese AAS asks respondents to indicate if they have been emotionally, physically or sexually abused (‘yes’ or ‘no’ option), whereas the Chinese CTS2 seeks to determine the severity of the abuse by measuring the frequency of the acts. Therefore, when the CTS2 is picking up more severe cases, the AAS is still identifying relatively less severe cases. This results in an increase in the number of false positives and a decrease in the number of false negatives. Nevertheless, with the downward trend of false negatives as shown in Figure 2, we are quite confident that severe cases of emotional abuse are not missed by the Chinese AAS. It may be deduced from this that women experiencing severe emotional abuse usually have little difficulty in identifying the abuse. To help reduce the false negatives for the milder forms of emotional abuse, we suggest that the wording of the emotional abuse items be refined by including more examples of milder forms of emotional abuse. In addition, by interviewing those in the present study who gave positive answers to emotional abuse items in the Chinese CTS2 but negative answers to emotional abuse items in the Chinese AAS, it may be possible to identify examples of emotional abuse that the Chinese AAS consistently fails to detect.

With respect to physical abuse, even though the Chinese AAS shows a downward trend in false negatives, the initial false-negative level (at 20% or above) requires attention (Figure 3). A likely explanation is that respondents may not realise that the identification of physical abuse is determined by the act itself and not by the injury resulting from the act. Hence, when answering the Chinese AAS, some may have given a (false) negative answer to physical abuse if no injury was caused by the abusive act. We suggest adding a statement in the Chinese AAS to remind respondents that physical abuse may or may not lead to injury. This may help detect milder forms of physical abuse before it escalates to a more serious level.

While some respondents may fail to identify milder forms of physical abuse, few of them have identified themselves as having been physically abused when the Chinese AAS is used but not when the Chinese CTS2 is used. This is reassuring and is confirmed by the low and consistent false positives as shown in Figure 3.

Measurement of emotional abuse is challenging, partly due to the lack of consensus on defining the construct.^23^ In the case of the Chinese population, the cultural norm of not disclosing family shame to outsiders^24^ makes it even more difficult to detect IPV. However, in the present study, some respondents were willing to disclose their IPV history. This is encouraging and is consistent with our findings in an earlier study.^14^ With the help of a validated Chinese AAS, the extent of emotional abuse in this hitherto ignored population can now be studied. This will advance our understanding of and scope for intervention to help Chinese women afflicted with the problem of emotional abuse in domestically violent relationships.

In this study, women who were in need of financial assistance were significantly more likely to be abused by their intimate partners. This is consistent with the findings that economic stress, low income and poverty are associated with a man abusing his partner.^1^,^25^,^26^ The age difference between the couple as a risk factor is also consistent with the findings of local studies.^16^,^27^ In recent years, there has been an influx of ‘younger’ women from Mainland China joining their ‘older’ husband in Hong Kong. The age difference may well be a symptom of marital instability leading to marital conflict. The identification of risk factors for IPV consistent with those reported in other studies lends further support to the use of the Chinese AAS as a tool for screening for IPV.

## Conclusion

The lack of a validated tool to screen Chinese women for IPV prompted us to undertake the present study. Using the Chinese CTS2 as the standard, the Chinese AAS has demonstrated satisfactory accuracy of measurement and holds promise of being a useful tool for screening for domestic violence among women in the Chinese population.

## References

[1] World Health Organization (2002). World Report on Violence and Health.

[2] American College of Obstetricians and Gynecologists (2002). Guidelines for Women’s Health Care.

[3] British Medical Association (1998). Domestic Violence: A Health Care Issue.

[4] Ramsay J, Richardson J, Carter YH, Davidson LL, Feder G (2002). Should health professionals screen women for domestic violence? Systematic review. BMJ.

[5] Nelson HD, Nygren P, Mclnerney Y, Klein J (2004). U.S. Preventive Services Task Force. Screening women and elderly adults for family and intimate partner violence: a review of the evidence for the U.S. Preventive services task force. Ann Intern Med.

[6] U.S. Preventive Services Task Force (2004). Screening for family and intimate partner violence: recommendation statement. Ann Intern Med.

[7] Parker B, McFarlane J (1991). Identifying and helping battered pregnant women. MCN Am J Matern Child Nurs.

[8] Campbell J, Furniss KK (2002). Violence Against Women: Identification, Screening and Management of Intimate Partner Violence.

[9] McFarlane J, Parker B, Soeken K, Bullock L (1992). Assessing for abuse during pregnancy. Severity and frequency of injuries and associated entry into prenatal care. JAMA.

[10] Soeken KL, McFarlane J, Parker B, Lominack MC, Campbell JC (1998). The abuse assessment screen: a clinical instrument to measure frequency, severity. and perpetrator of abuse against women. Empowering Survivors of Abuse: Health Care for Battered Women and Their Children.

[11] Reichenheim ME, Moraes CL (2004). Comparison between the abuse assessment screen and the revised conflict tactics scales for measuring physical violence during pregnancy. J Epidemiol Community Health.

[12] Leung WC, Leung TW, Lam YYJ, Ho PC (1999). The prevalence of domestic violence against pregnant women in a Chinese community. Int J Gynecol Obstet.

[13] Leung WC, Kung F, Lam J, Leung TW, Ho PC (2002). Domestic violence and postnatal depression in a Chinese community. Int J Gynecol Obstet.

[14] Tiwari A, Leung WC, Leung TW, Humphreys J, Parker B, Ho PC (2005). A randomised controlled trial of empowerment training for Chinese abused pregnant women in Hong Kong. BJOG.

[15] Straus MA, Hamby SL, Boney-McCoy S, Sugarman DB (1996). The revised conflict tactics scales (CTS2): development and preliminary psychometric data. J Fam Issues.

[16] Chan KL (2005). Study on Child Abuse and Spouse Battering: Report on Findings of Household Survey. [A Consultancy Study Commissioned By the SWD of the HKSAR Government].

[17] Maiuro RD, O’Leary KD, Maiuro RD (2001). Preface: sticks and stones may break my bones, but names will also hurt me: psychological abuse in domestically violent relationships. Psychological Abuse in Violent Domestic Relations.

[18] Straus MA (1995). Manual for the Conflict Tactics Scales.

[19] Zhou XH, Obuchowski NA, McClish DK (2002). Statistical Methods in Diagnostic Medicine.

[20] Jaeschke R, Guyatt GH, Sackett DL (1994). Users’ guides to medical literature. III. How to use an article about a diagnostic test. B. What are the results and will they help me in caring for my patients? The Evidence-based medicine working group. JAMA.

[21] The Government of Hong Kong Special Administrative Region (2001). Hong Kong 2001 Population Census Thematic Report.

[22] McFarlane J, Parker B, Soeken K, Silva C, Reed S (1999). Severity of abuse before and during pregnancy for African American, Hispanic, and Anglo women. J Nurse Midwifery.

[23] O’Leary KD, O’Leary KD, Maiuro RD (2001). Psychological abuse: a variable deserving critical attention in domestic violence. Psychological Abuse In Violent Domestic Relations.

[24] Tang CS, Lee A, Cheung FM, Cheung FM, Karlekar M, De Dios A, Vichit-Vadakan J, Quisumbing (1999). Violence against women in Hong Kong. Breaking the silence: Violence against women in Asia.

[25] Tjaden P, Thoennes N (2000). Full Report of the Prevalence, Incidence, and Consequences of Violence Against Women: Findings from the National Violence Against Women Survey.

[26] Vest JR, Catlin TK, Chen JJ, Brownson RC (2002). Multistate analysis of factors associated with intimate partner violence. Am J Prev Med.

[27] Tang CSK (1999). Wife abuse in Hong Kong Chinese families: a community survey. J Fam Violence.

